# The *PIRAGUA_atmos_analysis* and *PIRAGUA_hydro_analysis*: Water balance components data sets for the Pyrenees

**DOI:** 10.1016/j.dib.2026.113071

**Published:** 2026-07-17

**Authors:** Leticia Palazón, Pere Quintana-Seguí, Roger Clavera-Gispert, Anaïs Barella-Ortiz, Youen Grusson, Roxelane Cakir, Sabine Sauvage, José Miguel Sánchez-Pérez, Santiago Beguería

**Affiliations:** a Estación Experimental de Aula Dei (EEAD-CSIC), Zaragoza, Spain; b Observatori de l’Ebre (Universitat Ramon Llull-CSIC), Roquetes, Spain; c Centre de Recherche sur la Biodiversité et l'Environnement (CRBE), Université de Toulouse, CNRS, IRD, Toulouse INP, Toulouse, France

**Keywords:** Water resources, Water balance, Pyrenees, Spain, France, Andorre

## Abstract

The *PIRAGUA_atmos_analysis* and *PIRAGUA_hydro_analysis* datasets have been developed to support a comprehensive assessment of current water resources across the Pyrenees region, encompassing Spain, France, and Andorra. *PIRAGUA_atmos_analysis* is a daily, gridded dataset of near-surface meteorological variables designed to serve as input for hydrological and land-surface models. It is based on the SAFRAN reanalysis framework and assimilates observational data from both the Spanish and French meteorological agencies, covering the hydrological years from September 1979 to August 2014. *PIRAGUA_hydro_analysis* provides monthly water balance components derived from the SASER and SWAT hydrological models for the 1981–2010 period, driven by *PIRAGUA_atmos_analysis* forcing. This article documents the development of both datasets and illustrates their potential applications. Alongside the datasets, we provide example R scripts for data exploration, an interactive visualization and analysis platform, and access through a dedicated geo-server. These resources aim to foster transparency, reproducibility, and informed decision-making in regional water management and planning.

Specifications Table

**Table:** 

Subject	Environmental Science / Climatology; Environmental Science / Hydrology and Water Quality.
Specific subject area	Water balance, Water resources.
Type of data	Plain text data tables, GIS shapefiles, and R code.
Data collection	The data sets are based on the assimilation of meteorological observations from the Spanish and French meteorological agencies using the SAFRAN methodology, and on the simulation of the main water balance components obtained using SASER and SWAT hydrological models.
Data source location	Region: Pyrenees.Countries: Spain, Andorre and France.Coordinates (latitude/longitude): covers the area limited by 40.615/−4.536 and 45.626/4.505, according to the coordinate system WGS84 (EPSG: 4326).
Data accessibility	*Repository:*https://digital.csic.es/*. Data identification number (DOI):* • PIRAGUA_atmos_analysis: 10.20350/digitalCSIC/14,665; • PIRAGUA_hydro_analysis: 10.20350/digitalCSIC/14,667; • PIRAGUA_recipes: 10.20350/digitalCSIC/16,381. Interactive data explorer: https://opcc-ctp.org/geoportal.
Related research article	*Water balance components of the Pyrenees: a 30-year modeling study in a transboundary context [**1**].*

## 1 Value of the Data

• *PIRAGUA_atmos_analysis* is, to our knowledge, the first seamless, high-resolution (2.5 km) meteorological reanalysis spanning the entire Pyrenees across the Spanish–French–Andorran border, harmonising observations from the Spanish (AEMET) and French (Météo-France) agencies within a single SAFRAN framework. It removes the discontinuities that arise when national products are mosaicked, providing consistent forcing for transboundary hydrological and land-surface modelling.
• The paired *PIRAGUA_hydro_analysis* outputs are generated by two structurally independent hydrological models: SASER (fully distributed, physically based, uncalibrated) and SWAT (semi-distributed, conceptual, calibrated), driven by the same forcing. The spread between them provides reusers with a direct, ready-to-use estimate of the structural (epistemic) uncertainty of each water-balance component, rather than a single deterministic value.
• The datasets cover a continuous 30-year baseline (1981–2010) consistent with World Meteorological Organization climate-normal conventions, enabling robust analysis of long-term means, interannual variability, and trends, and serving as a reference against which recent observations and future climate- or land-use-change scenarios can be compared.
• Companion R scripts (*PIRAGUA_recipes*) and the interactive geoportal of the Pyrenean Climate Change Observatory (OPCC), with Web Map Service access, substantially lower the barrier to reuse, allowing non-specialist users to locate, visualise, map, and extract the data without dedicated modelling expertise.
• The data support water-resource planning, environmental-flow and reservoir assessment, and sector-specific impact studies (irrigated agriculture, hydropower, snowpack dynamics) across France, Spain, and Andorra; *PIRAGUA_atmos_analysis* can additionally serve as meteorological forcing for any environmental or land-surface model over the full Ebro and Adour–Garonne drainage basins.

## 2 Background

**Fig. 1:**
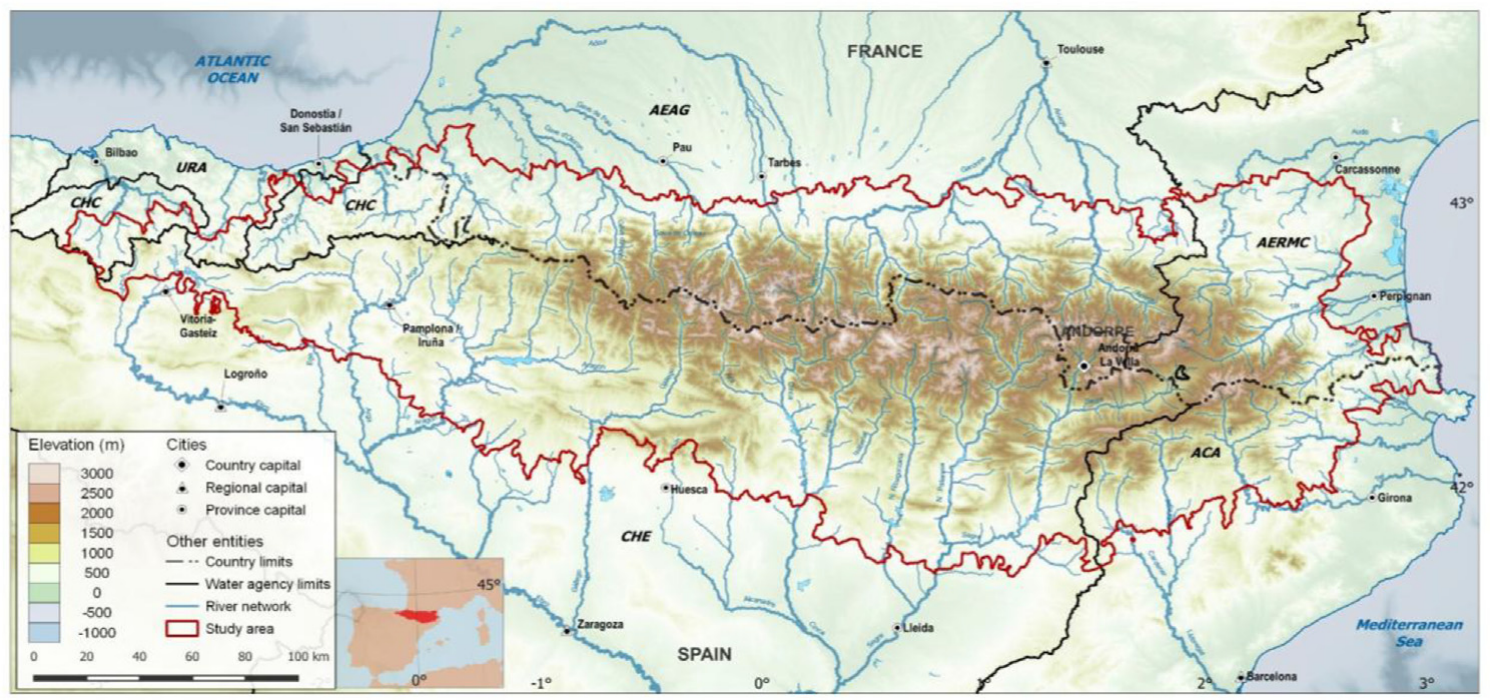
Location and main features of the study area.

The Pyrenees mountain range is a primary freshwater source for southwestern Europe, spanning the territories of France, Spain, and Andorra (Fig. 1). To support integrated water resource analysis in this region, we compiled high-resolution atmospheric and hydrological datasets based on the best available observations and modelling tools.

The resulting collection enables a consistent reconstruction of the regional water balance under historical climate conditions (1981–2010), and allows for a detailed evaluation of the uncertainties inherent to hydrological modelling. Two hydrological models were used: SASER, a fully distributed physical model, and SWAT, a semi-distributed conceptual model.

In this article, we describe the development and structure of the datasets and showcase several examples of their use. Additionally, we provide R code for reproducible analysis, an interactive web-based visualization tool, and a dedicated geo-server for easy data access.

## 3 Data Description

Meteorological and hydrological analysis: the *PIRAGUA_atmos_analysis* and *PIRAGUA_hydro_analysis* data sets.

*PIRAGUA_atmos_analysis* is a high-resolution gridded dataset of near-surface meteorological variables developed using the SAFRAN reanalysis methodology, and is specifically designed to serve as input for hydrological and land-surface modelling. It spans 35 hydrological years (September–August), from 1 September 1979 to 31 August 2014 (12,784 daily time steps). This full record is longer than the sub-period used to force the hydrological simulations described below (whose common analysis window is 1981–2010). The grid is referenced in the UTM projection (ETRS89 datum, zone 30 N; EPSG:25,830) and spans 370,000–1085,000 m easting (286 columns) and 4465,000–5080,000 m northing (246 rows); a geographic bounding box is given in the Specifications table. The spatial resolution is 2.5 × 2.5 km and the temporal resolution is daily.

The dataset is distributed as one compressed file per variable. Upon extraction, each contains a single NetCDF file (.nc; CF-1.6 conventions) holding the complete daily time series for that variable as a 3-D array (time × y × x); the two static fields (Surface_Pressure and Terrain_Elevation) are stored as 2-D arrays (y × x) without a time dimension. Missing values are coded as −999. Files follow the naming convention PIR1-<period>-<Variable>.nc (for example, PIR1–19,792,014-Liquid_Precipitation.nc). The complete list of variables, with their internal names and units, is given in Table 1.

**Table 1:** Variables in the *PIRAGUA_atmos_analysis* data set.

Name	Description	Unit
ET0_PM	Daily reference (potential) evapotranspiration, Penman–Monteith method	kg/m² (mm)
Liquid_Precipitation	Total daily liquid precipitation	kg/m² (mm)
Solid_Precipitation	Total daily solid precipitation	kg/m² (mm)
Relative_Humidity_max	Maximum daily relative humidity	%
Relative_Humidity_mean	Mean daily relative humidity	%
Relative_Humidity_min	Minimum daily relative humidity	%
Temperature_max	Maximum daily temperature	°C
Temperature_mean	Mean daily temperature	°C
Temperature_min	Minimum daily temperature	°C
Wind_Speed	Mean daily wind speed	m/s
Surface_Pressure	Surface atmospheric pressure (static field, no time dimension)	hPa
Terrain_Elevation	Terrain elevation of the grid cell (static field, no time dimension)	m

**Fig. 2:**
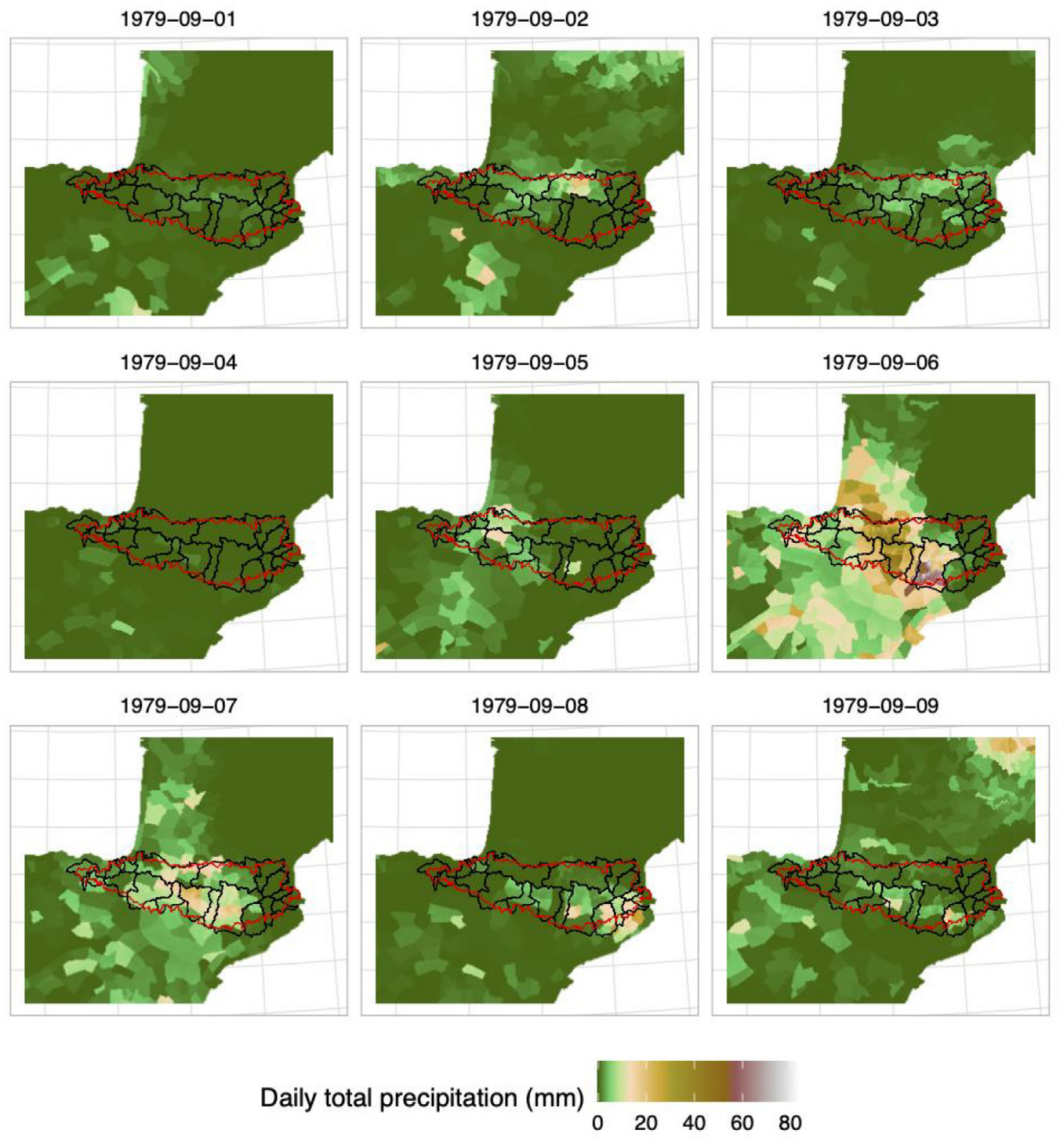
Example of *PIRAGUA_atmos_analysis*. Daily total precipitation during the first nine days of 1979.

NetCDF files can be accessed using a variety of free and open-source tools such as Panoply or QGIS, or programmatically through R or Python using dedicated libraries. An example time series of daily precipitation maps derived from *PIRAGUA_atmos_analysis*, generated using the companion R code package (*PIRAGUA_recipes*), is shown in Fig. 2.

Importantly, the spatial extent of *PIRAGUA_atmos_analysis* extends well beyond the Pyrenees, encompassing the full drainage basins of the Ebro and Adour–Garonne rivers. This facilitates the application of the dataset for hydrological simulations over a broader region.

The *PIRAGUA_hydro_analysis* dataset provides geospatial information on simulated water balance components and river discharge from 1981 to 2010, derived from the SASER and SWAT hydrological models. Table 2 details the included variables. The data is available at annual and monthly resolutions and covers various spatial scales: hydrographic sub-basins, basins, Andorra, Spain, France, and the entire Pyrenees range.

**Table 2:** Variables in the *PIRAGUA_hydro_analysis* data set.

Name	Description	Unit
PRECIP	Total precipitation (liquid + solid) over the month / year	mm
SNOFALL	Snowfall (solid precipitation) over the month / year	mm
SNOMELT	Snowmelt over the month / year	mm
TMP_AV	Mean daily temperature, averaged over the month / year	°C
TMP_MX	Mean daily maximum temperature, averaged over the month / year	°C
TMP_MN	Mean daily minimum temperature, averaged over the month / year	°C
ET	Actual evapotranspiration over the month / year	mm
PET	Potential evapotranspiration over the month / year	mm
ET_PET	Ratio of actual to potential evapotranspiration	mm/mm
SW	Mean soil-water storage over the month / year	mm
SWE	Mean snowpack (snow water equivalent) over the month / year	mm
DA_RCHG	Deep aquifer recharge over the month / year	mm
WYLD	Total water yield over the month / year	mm
SNO_WYLD	Snowmelt contribution to total water yield	mm/mm
CONTRIB_CMS	Mean river discharge at the sub-basin outlet	m³/s
CONTRIB_HM3	Mean water contribution at the sub-basin outlet	Mm³

*PIRAGUA_hydro_analysis* is distributed as four files: two plain-text data tables in comma-separated-values (.csv) format (*PIRAGUA_hydro_analysis_s-mode*.csv and *PIRAGUA_hydro_analysis_t-mode*.csv), a compressed GIS archive (gis.zip) containing the shapefiles of the spatial units, and a documentation file (*PIRAGUA_hydro_analysis*.md) that describes every field and the unit conversions. A shared text identifier field, *id*, links each row of the data tables to the corresponding polygon or reach in the shapefiles, enabling mapping in GIS software such as QGIS or in programming languages such as Python and R.

The *PIRAGUA_hydro_analysis_s-mode* table reports, for every spatial unit, the average annual and monthly values of each water-balance component over 1981–2010, whereas *PIRAGUA_hydro_analysis_t-mode* contains the corresponding complete annual and monthly time series. The GIS archive contains four shapefiles: the study-area boundary (pyrenees.shp), the river basins (basins.shp), the sub-basins (subbasins.shp), and the river-network reaches (reaches.shp).

**Fig. 3:**
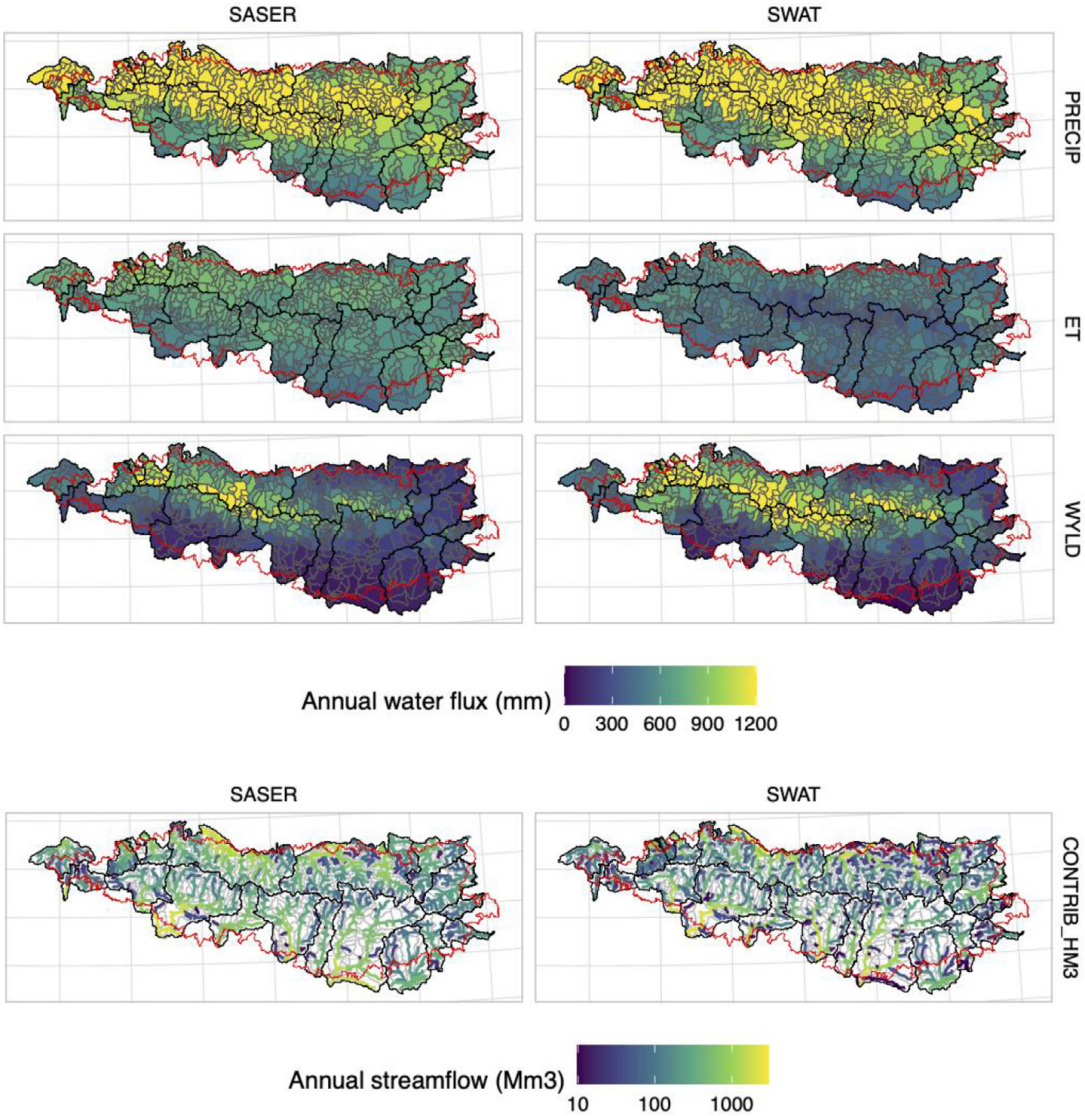
Examples of *PIRAGUA_hydro_analysis_s-mode*. Top: 1981–2010 mean annual water fluxes (precipitation, evapotranspiration and water yield). Bottom: 1981–2010 mean annual streamflow. Comparison between two models (SASER and SWAT), at the sub-basin scale.

The *PIRAGUA_hydro_analysis_s-mode* file, thus, presents average water balance components and their spatial variability. For instance, Fig. 3 (created using *PIRAGUA_recipes*) illustrates mean annual water fluxes and streamflow at the sub-basin level. Similar maps for other water balance components can be generated, including at a monthly scale.

**Fig. 4:**
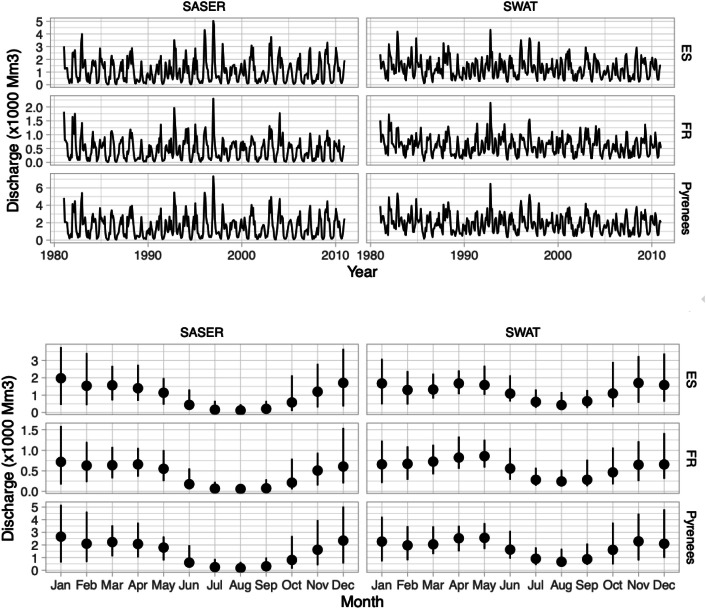
Examples of *PIRAGUA_hydro_analysis_t-mode*. Top: time series of aggregated monthly discharge at the Pyrenees river outlets. Bottom: monthly mean discharge climatologies over the 1981–2010 period: median (dots) and 80 percent range (vertical lines). Results are shown per country (ES: Spain; FR: France), and for the whole area (Pyrenees).

The *PIRAGUA_hydro_analysis_t-mode* dataset enables the analysis of hydrological component time series for both models. This functionality facilitates direct time series comparisons (Fig. 4, top panel) and the creation of insightful climatologies (Fig. 4, bottom panel).

### 3.1 PIRAGUA_recipes

The *PIRAGUA_recipes* repository contains an R project (RStudio) with a set of worked, step-by-step examples for exploring the *PIRAGUA_atmos_analysis* and *PIRAGUA_hydro_analysis* datasets, including the generation of several of the maps and plots shown in this article. These scripts are illustrative of typical analysis workflows; they are not required to read or access the data, which are distributed in standard, self-describing formats (NetCDF and CSV). After decompressing *PIRAGUA_recipes*.zip, the examples are run by opening *PIRAGUA_recipes*.Rproj in RStudio and executing the notebook R/*PIRAGUA_recipes.Rmd*.

### 3.2 Interactive geoportal

The PIRAGUA datasets are also accessible through the interactive geoportal of the Pyrenean Climate Change Observatory (OPCC), a collaborative effort involving the governments of Aragon, Catalonia, Basque Country, and Navarre (Spain), Nouvelle-Aquitaine and Occitanie (France), and Andorra (https://www.opcc-ctp.org/es/geoportal). This user-friendly tool offers the public essential data regarding climate change impacts in the Pyrenees. It provides direct access to the datasets, allowing for map creation and some analytical functions. The platform is accessible via a web browser or through GIS software using the Web Map Service (WMS) protocol.

**Fig. 5:**
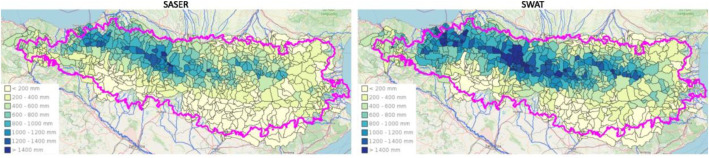
Water balance geoportal examples: average annual water yield for the assessed period (1981–2010) from SASER and SWAT.

The *PIRAGUA_hydro_analysis* data sets are included by maps in the water balance sub-menu of the “Water Resources” thematic area section of the Geoportal. *PIRAGUA_hydro_analysis* is shown in the sub-menu related to its simulation period (1981–2010), with averaged annual values by water balance variable and hydrological model (Fig. 5). All of these maps could be downloaded from the Geoportal. In this way, the geoportal complements direct download of the data files described above: it lets users visualise and query the spatial data, and access them from GIS software through the Web Map Service (WMS) protocol, without downloading or decompressing the full datasets.

## 4 Materials and Methods

### 4.1 Meteorological analysis

Meteorological variables are a fundamental input for hydrological modelling. Therefore, as an initial step we constructed *PIRAGUA_atmos_analysis*, a gridded data set of critical meteorological variables using SAFRAN (*Système d’Analyse Fournissant des Renseignements Atmosphériques à la Neige*; [2]). This tool synthesizes screen-level meteorological data, such as precipitation, temperature, wind speed, and relative humidity, by integrating meteorological model outputs with empirical observations through an optimal interpolation algorithm.

SAFRAN operates via a dual-stage process. Initially, it employs an optimal interpolation algorithm to approximate the values of the targeted variables across meteorologically homogenous zones (defined as areas approximately 1000 km² in size with uniform climatic conditions) at various altitudes (one tier every 300 m). This system ingests daily precipitation measurements and six-hourly updates for other variables, producing hourly data sets through various temporal interpolation techniques. Subsequently, these values are interpolated onto a regular grid, considering the altitudinal variations of each grid point. Thus, within a single zone, variations in variable values, such as temperature, arise solely from differences in altitude [3].

For *PIRAGUA_atmos_analysis*, we merged the French and Spanish implementations of SAFRAN [4]. In this configuration, SAFRAN combines a first-guess field from the ERA5 reanalysis with screen-level observations from the Spanish (AEMET) and French (Météo-France) meteorological agencies through an optimal interpolation algorithm; the merging of the two national domains and the new interpolation onto a unified 2.5 × 2.5 km grid were carried out at the Observatori de l'Ebre. This revised grid supports the hydrological models and encompasses the full study area, including all basins that drain the Pyrenean range.

### 4.2 Hydrological analysis

Hydrological analysis facilitated the simulation of water storages (snowpack, soil moisture) and water flows (evaporation, transpiration, runoff, drainage). Calculated spatially, these simulations offer a thorough assessment of water balance elements and their changes over time across the study region. The resulting data are stored in the *PIRAGUA_hydro_analysis* dataset, organized by varying temporal and spatial resolutions.

For the hydrological analysis we utilized two distinct hydrological models: the fully distributed land-surface model chain SASER and the semi-distributed hydrological model SWAT. Brief introductions to these models are provided below. For a comprehensive understanding of their theory and implementation, please refer to their respective websites: http://www.umr-cnrm.fr/surfex/ (last accessed: 19/06/2026) and https://swat.tamu.edu/ (last accessed: 19/06/2026).

### 4.3 SASER

SASER (SAfran-Surfex-Eaudyssée-Rapid) is a complete hydro-meteorological modelling platform that utilizes SAFRAN for atmospheric forcing, SURFEX to assess the water and energy balances of the terrestrial surface, and Eaudyssée and RAPID to convert SURFEX outputs on runoff and drainage into flow rates, subsequently routing these through the hydrographic network [5]. This integration facilitates a comprehensive simulation of the continental hydrological cycle.

SURFEX (SURFace EXternalisée, version 8.1), developed by Météo France, is a physically-based, fully-distributed land surface model that calculates the hydrologic and energy balances on continental surfaces using a uniform grid [6]. The model employs the ISBA (Interaction Sol-Biosphère-Atmosphère) land surface scheme for natural soils, specifically utilizing the multi-layer diffusion variant, ISBA-DIF [7], which computes surface energy and water budgets over natural terrains. While SURFEX itself does not include a native routing module, it is complemented by RAPID (Routing Application for Parallel ComputatIon of Discharge; [8]), a routing model that effectively channels total runoff (from surface and subsurface) across SURFEX grid cells to river cells using an isochrony algorithm. RAPID then employs a matrix-based version of the Muskingum method to determine flow and water volume for each segment of a river network. It is crucial to note that this setup does not simulate human alterations such as dams, canals, or irrigation systems, and hence provides an estimation of the system in its natural state.

Eau-dyssée serves as a framework that intricately couples hydrological models, in this instance SURFEX and RAPID, to manage disparate spatial and temporal scales while facilitating the necessary exchange of relevant variables between the two [9].

### 4.4 SWAT

**Fig. 6:**
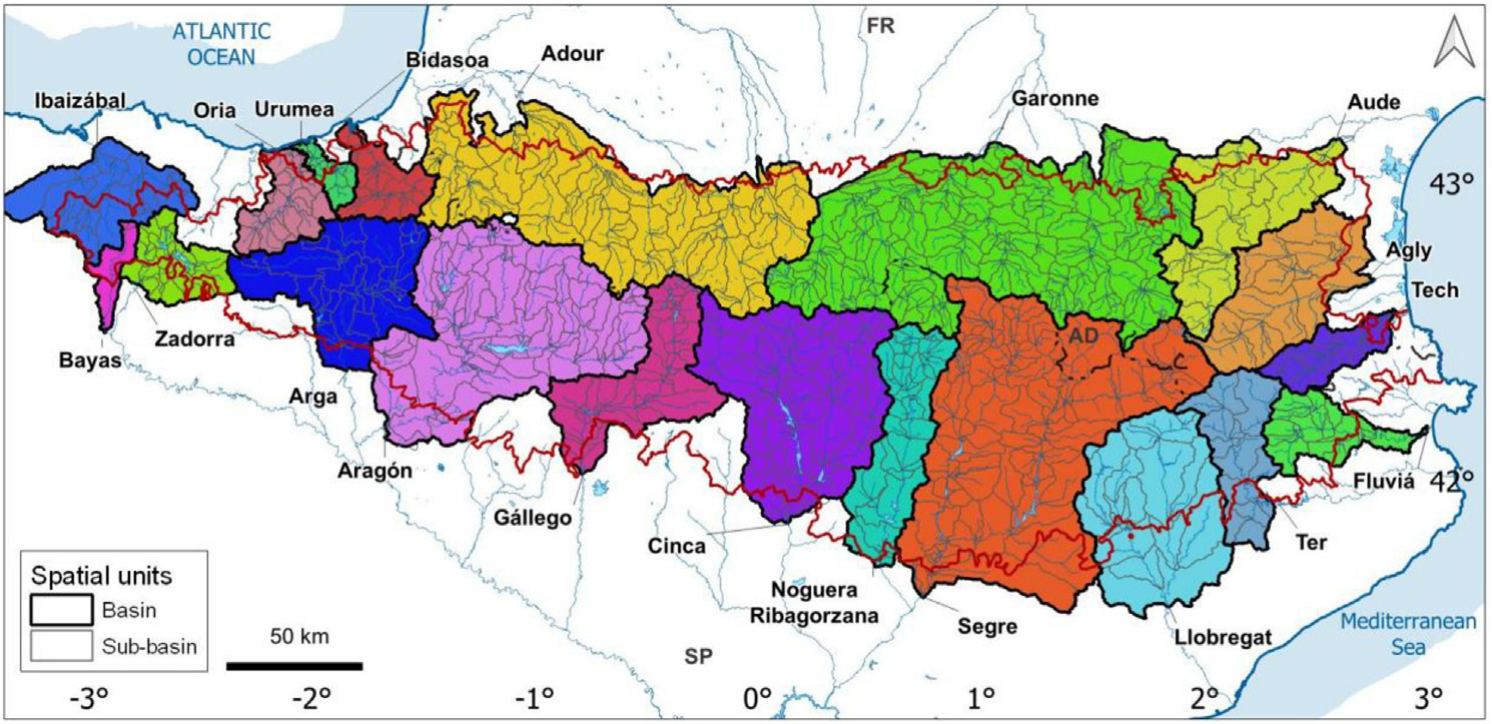
Hydrological simulation domains: hydrographic basins and sub-basins.

SWAT (Soil and Water Assessment Tool; [10]) is a semi-distributed, basin-scale, time-continuous hydrological model endorsed by the USDA Agricultural Research Service. Recent reviews summarise its performance and limitations across a wide range of applications [11], and a completely restructured version, SWAT+, has since been released [12]. For the purposes of this study, the area of interest was segmented into 20 distinct basins, as illustrated in Fig. 6. Each basin was further subdivided into sub-basins, and these into hydrological response units (HRUs) using SWAT (version 10.4). HRUs are defined by their uniform land use, soil types, and slope categories, and are the basic simulation units in the model.

Given its conceptual framework, SWAT necessitates comprehensive calibration and validation of critical parameters. The designated periods for these processes were: calibration from 1986 to 2005, and validation from 2006 to 2013. Calibration was executed in two phases: an initial calibration integrating all selected gauge stations to stabilize sensitive parameters across each basin, followed by a targeted recalibration for the upper catchments at gauge stations where the initial simulations were suboptimal, thus refining the model to reflect local hydrological nuances in headwater areas.

Once calibrated and validated, the established parameters were applied retrospectively for the entire simulation periods from 1981 to 2010. To accommodate SWAT’s requirement for an initialization phase, or ``warm-up'', the climatic input data from the initial 5 years period (i.e. 1980 to 1985) was duplicated and assigned as the previous years of simulation (i.e. 1975 to 1979), thereby ensuring the system was primed for the full simulation periods of interest.

### 4.5 Input data

Additional data were necessary to characterize different inputs such as topography, soil types and land use and land cover, and in the case of SWAT, streamflow data were collected to calibrate and validate the model projects. These data were collected from various sources, as required by the hydrological models. Importantly, land cover and soil characteristics were assumed to remain constant over the simulation period. This assumption, while common in hydrological modelling, has implications for the interpretation of long-term trends and interannual variability, which we discuss later in the results and discussion sections.

A comprehensive summary of the additional input datasets (including sources, spatial resolution, and preprocessing steps) is provided in Table 3.

**Table 3:** Additional input data sources.

Model	Inputs	Source	Grid resolution and preprocessing steps
SASER			
	Topography	GTOPO-30 data set [13]	30×30m
	Physiography	ECOCLIMAP II [14]	1 × 1km
	Hydrology	HydroSheds [15]	500×500m
	Radiation fluxes	Downward infrared radiation ERA-5 [16]	31×31km
SWAT			
	Topography	SRTM90 [17]	90×90m
		EuDEM v 1.1. (https://land.copernicus.eu)	25×25m
	Land use/cover	Corine Land Cover of 2012 [18]	250×250m Resampled to 100×100m Completed with Andorran land cover information
	Soil types	Harmonized World Soil Database [19]	1 × 1km Resampled 100×100m
	Sub-basins definition	Superficial water body basin delineations (Directive 2000/60/EC)	-

### 4.6 Delimitation of hydrological spatial units

Delimitation of the hydrographic network and main spatial units (basin and sub-basins) used in the hydrological modelling and to construct the *PIRAGUA_hydro_analysis* data set was carried out using the ArcGIS SWAT interface [20]. For almost all of the defined basins (Fig. 6), delimitation was based on the SRTM90 digital elevation model with a 90×90 m spatial resolution [17]. This dataset provided a consistent basis for identifying drainage patterns and watershed boundaries across the mountainous terrain of the Pyrenees. However, in certain small coastal catchments, such as the Agly and Tech basins, characterized by low slope gradients, the SRTM90 resolution was insufficient to accurately capture drainage divides and streamflow direction. To improve the realism of the hydrographic representation in these flatter areas, we incorporated the EU-DEM version 1.1 (https://land.copernicus.eu), which offers a finer spatial resolution of approximately 25 × 25 m. This allowed for improved definition of flow paths and sub-basin structure, ensuring consistent hydrological routing and better spatial representation across the full range of topographic conditions in the study area.

The subdivision of basins into sub-basins was aligned with the superficial water body basin delineations specified by the European Water Framework Directive (Directive 2000/60/EC), with some adjustments made to ensure alignment with the locations of gauge stations of interest for model calibration, validation, and analysis, yielding 822 sub-basins. Finally, the main spatial units delimited were used as spatial basis for both hydrological model results and are the following (basin name, and its number of sub-basins in parentheses): Adour (67), Agly (14), Aragón (84), Arga (41), Aude (20), Bayas (4), Bidasoa (17), Cinca (67), Fluviá (21), Gállego (43), Garonne (174), Ibaizábal (28), Llobregat (20), Noguera-Ribagorzana (37), Oria (21), Segre (92), Tech (25), Ter (13), Urumea (14) and Zadorra (20).

### 4.7 Calibration and validation

Observed streamflow data were compiled from hydrological authorities across the transboundary Pyrenean region, including Confederación Hidrográfica del Cantábrico (CHC), Agencia Vasca del Agua / Ur Agentzia (URA), Confederación Hidrográfica del Ebro (CHE), Agència Catalana de l’Aigua (ACA), Agence de l’eau Adour-Garonne (AEAG), and Agence de l’eau Rhône-Méditerranée-Corse (AERMC). For France, data were obtained through HydroPortail (https://hydro.eaufrance.fr/, EAUFR.), which consolidates measurements from multiple regional agencies.

Station selection prioritized both data quality and the representativeness of natural hydrological conditions. Inclusion criteria were: (i) location in headwater catchments within the Pyrenees; (ii) minimal anthropogenic influence (e.g., absence of large dams or withdrawals); (iii) unaltered seasonal flow regimes, particularly stable summer low flows; (iv) absence of abrupt discontinuities in the records; (v) continuous coverage of at least 20 years with no missing hydrological years; and (vi) records extending up to 2013. Applying these filters, 87 gauging stations were retained (Supplementary Table 1 and Supplementary Figure S1), spanning the period September 1980–August 2013. Hydrological years were defined from September to August, with seasons grouped as SON (autumn), DJF (winter), MAM (spring), and JJA (summer).

Calibration was applied only to SWAT, while SASER was run in a fully distributed, uncalibrated mode. SWAT calibration targeted streamflow and involved parameters regulating soil water storage, vegetation evapotranspiration, shallow aquifer recharge and return flow, and deep aquifer percolation (treated as a net loss). Parameter selection and value ranges were informed by prior hydrological studies in the same area [1]. The procedure followed a two-phase strategy: (i) calibration against observed daily discharges at selected stations, and (ii) targeted refinements in upstream catchments where performance was initially suboptimal. The calibration period spanned 1986–2005, with independent validation for 2006–2013. [1]).

The primary calibration criterion was the Kling–Gupta Efficiency (KGE; [21]), optimized using the Sequential Uncertainty Fitting algorithm (SUFI-2) within the SWAT-CUP (version 5.1.6, 2012) framework [22]. Model skill during validation was assessed with KGE alongside percent bias (PBIAS), Nash–Sutcliffe Efficiency (NSE), and the coefficient of determination (R²). Metrics were computed at both annual and seasonal scales, though we report annual summaries after de-seasonalisation.

**Fig. 7:**
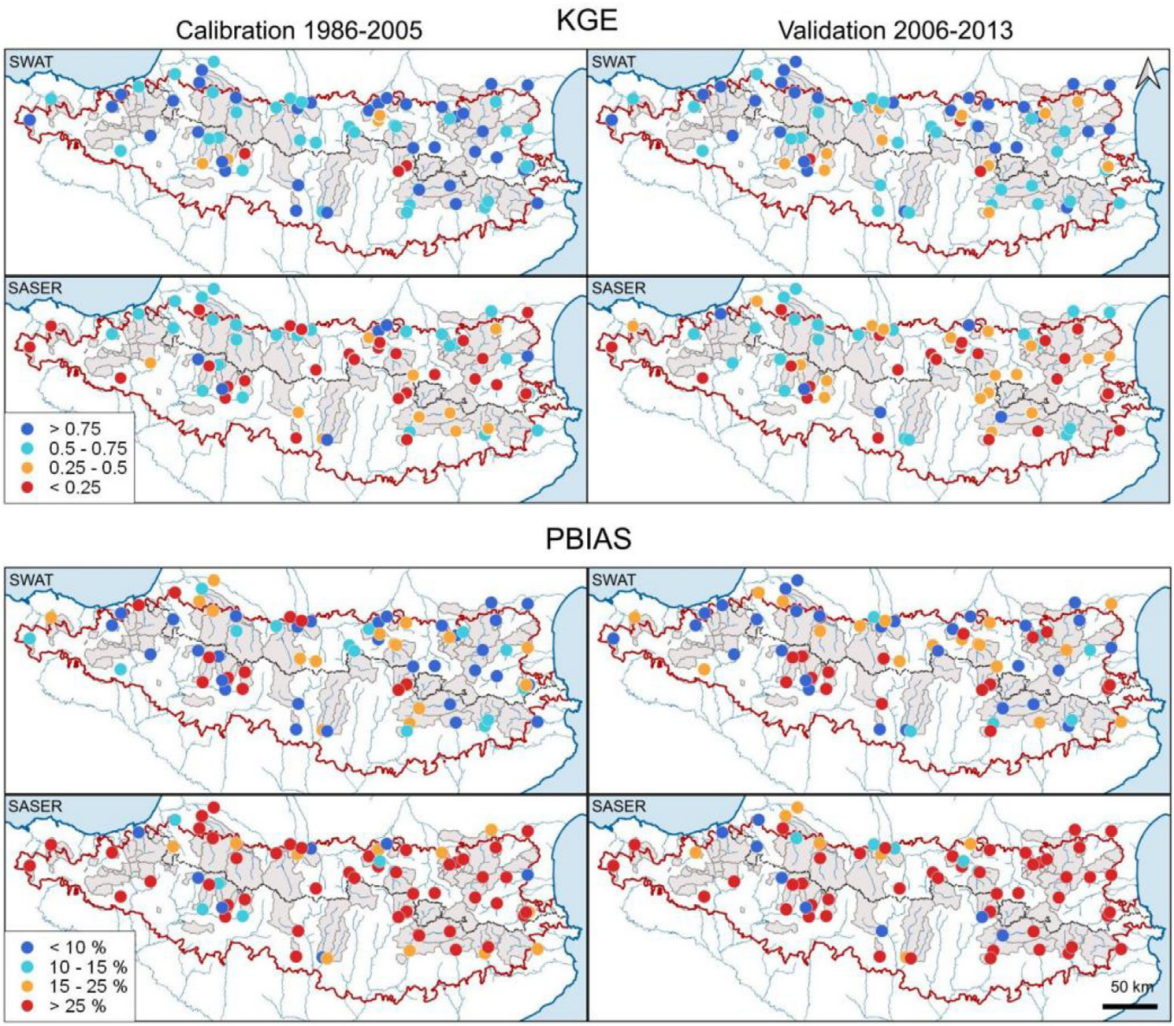
Model performance for calibration and validation periods: KGE and PBIAS values at selected gauging stations.

Across the 87 stations, the calibrated SWAT model achieved good and stable skill (median KGE ≈ 0.75 in calibration and ≈ 0.71 in validation, with most stations within ±15 % bias), whereas the uncalibrated SASER reproduced the main seasonal and interannual patterns at lower efficiency (median KGE ≈ 0.45) and larger bias, without a clear regional pattern. These summary statistics are reported so that reusers can gauge the reliability of the discharge data; the complete per-station results, their spatial distribution, and an independent evaluation against MODIS evapotranspiration and snow-cover products are provided in the companion research article [1]. Fig. 7 summarises model performance at the selected gauging stations.

## Limitations

As model-derived products, the PIRAGUA datasets are subject to several sources of uncertainty that users should weigh when interpreting and reusing the data. The most relevant ones are summarised below; a full, quantitative assessment of model performance and of the structural (epistemic) uncertainty between the two hydrological models is provided in the companion research article [1].

**Propagation of meteorological-forcing error.** *PIRAGUA_atmos_analysis* is itself a reanalysis that blends a first-guess atmospheric model (ERA5) with station observations through optimal interpolation. Its accuracy is therefore constrained by the density and quality of the underlying observation network (which is sparse at high elevations) and by the merging of the Spanish and French SAFRAN implementations. Biases in the forcing, particularly in high-altitude precipitation and the rain/snow partition, propagate into all hydrological outputs.

**Static land cover and soil.** Both hydrological models were run with land cover and soil properties held constant over the simulation period. Real changes in vegetation, land use, and soils are thus not represented, which affects the interpretation of long-term trends and interannual variability, especially for evapotranspiration and water yield.

**Naturalised regime.** The simulations do not include dams, reservoirs, diversions, or abstractions. The discharge and water-balance values therefore describe a naturalised system and will differ from observed flows in regulated reaches; they are best suited to characterising baseline resources rather than managed operations.

**Structural divergence between models.** SASER is a fully distributed, physically based chain run without calibration, whereas SWAT is a semi-distributed conceptual model calibrated to observed discharge. The two bracket a plausible range rather than converging on a single value: they agree closely on large-scale climatic gradients but diverge in the magnitude and timing of evapotranspiration, snowmelt, and groundwater fluxes, a contrast also documented for Pyrenean streamflow using a multi-model approach [23]. Calibration improves SWAT's skill at gauged outlets but does not guarantee the realism of internal fluxes, while SASER's uncalibrated formulation preserves physical consistency at lower discharge skill; subsequent developments have since improved SASER's low-flow simulation [24]. Users should therefore treat the spread between the two models as an estimate of structural uncertainty and avoid relying on either model alone for any single component.

**Temporal scope.** The water-balance datasets describe the 1981–2010 baseline and do not capture the accelerated warming and snowpack decline documented in the Pyrenees since 2010 (e.g., [25] and [26]). They are intended as a spatially consistent historical reference against which recent observations and future scenarios can be assessed.

## Ethics Statement

The authors have read and followed the ethical requirements for publication in Data in Brief, and confirm that the current work does not involve human subjects, animal experiments, or any data collected from social media platforms.

## CRediT Author Statement

**Santiago Beguería:** Funding acquisition, Conceptualization, Software, Formal Analysis, Writing - Original Draft; **Leticia Palazón:** Methodology (SWAT simulations Spain and Andorra), Formal Analysis, Writing - Original Draft; **Anaïs Barella:** Methodology (SASER simulations); **Roxelane Cakir:** Methodology (SWAT simulations, France). **Youen Grusson:** Methodology (SWAT simulations, France). **Pere Quintana-Seguí:** Funding acquisition, Conceptualization, Methodology (SAFRAN analysis and scenarios, SASER simulations), Formal Analysis, Writing - Original Draft. **José Miguel Sánchez-Pérez:** Funding acquisition, Validation (SWAT simulations, France). **Sabine Sauvage:** Methodology and Validation (SWAT simulations, France). **Jean-Philippe Vidal:** Methodology (SAFRAN scenarios). **Pierre Le Cointe:** Methodology (SAFRAN scenarios).

## Funding Sources

This work was supported by the project EFA210/16/PIRAGUA co-founded by the European Regional Development Fund (ERDF) through the Interreg V Spain-France-Andorre Programme (POCTEFA 2014–2020) of the European Union.

## Data Availability

digitalCSICPIRAGUA_hydro_analysis (Original data).

## Appendix Supplementary materials

Image, application 1

## References

[1] Beguería S., Palazón L., Quintana-Seguí P., Clavera-Gispert R., Barella-Ortiz A., Cakir R., Grusson Y., Sauvage S., Sánchez-Pérez J.M. (2026). Water balance components of the Pyrenees: a 30-year modelling study in a transboundary context. J. Hydrol..

[2] Durand Y., Giraud G., Brun E., Merindol L., Martin E. (1999). A computer-based system simulating snowpack structures as a tool for regional avalanche forecasting. J. Glaciol..

[3] Quintana-Seguí P., Le Moigne P., Durand Y., Martin E., Habets F., Baillon M., Canellas C., Franchisteguy L., Morel S. (2008). Analysis of near-surface atmospheric variables: validation of the SAFRAN analysis over France. J. Appl. Meteorol. Clim..

[4] Quintana-Seguí P., Turco M., Herrera S., Miguez-Macho G. (2017). Validation of a new SAFRAN-based gridded precipitation product for Spain and comparisons to Spain02 and ERA-interim. Hydrol. Earth Syst. Sci..

[5] Gaona J., Quintana-Seguí P., Escorihuela M.J., Boone A., Llasat M.C. (2022). Interactions between precipitation, evapotranspiration and soil moisture-based indices to characterize drought with high-resolution remote sensing and land-surface model data. Nat. Hazard. Earth Syst. Sci. Discuss..

[6] Le Moigne P., Besson F., Martin E., Boé J., Boone A., Decharme B., Etchevers P., Faroux S., Habets F., Lafaysse M., Leroux D., Rousset-Regimbeau F. (2020). The latest improvements with SURFEX v8.0 of the Safran-Isba-Modcou hydrometeorological model for France. Geos. Model Dev..

[7] Habets F., Boone A., Noilhan J. (2003). Simulation of a Scandinavian basin using the diffusion transfer version of ISBA. Glob. Planet. Chang..

[8] C.H. David, 2013. RAPID v1.4.0 (v1.4.0). Zenodo.

[9] Flipo N., Monteil C., Poulin M., de Fouquet C., Krimissa M. (2012). Hybrid fitting of a hydrosystem model: long-term insight into the Beauce aquifer functioning (France). Water Resour. Res..

[10] Douglas-Mankin K.R., Srinivasan R., Arnold J.G. (2010). Soil and water assessment tool (SWAT) model: current developments and applications. Trans. ASABE.

[11] Tan M.L., Gassman P.W., Yang X., Haywood J. (2020). A review of SWAT applications, performance and future needs for simulation of hydro-climatic extremes. Adv. Water Resour..

[12] Bieger K., Arnold J.G., Rathjens H., White M.J., Bosch D.D., Allen P.M., Volk M., Srinivasan R. (2017). Introduction to SWAT+, a completely restructured version of the soil and water assessment tool. JAWRA J. Am. Water Resour. Assoc..

[13] Earth Resources Observation and Science (EROS) Center, 2017. Global 30 arc-Second elevation (GTOPO30). [Work]. U.S. Geological Survey. 10.5066/f7df6pqs.

[14] Faroux S., Kaptue Tchuente A.T., Roujean J.-L., Masson V., Martin E., Moigne P.L. (2013). ECOCLIMAP-II/Europe: a twofold database of ecosystems and surface parameters at 1 km resolution based on satellite information for use in land surface, meteorological and climate models. Geosci. Model Dev..

[15] Lehner B., Verdin K., Jarvis A. (2008). New global hydrography derived from spaceborne elevation data. Eos Trans. Am. Geophys. Union.

[16] Hersbach H., Bell B., Berrisford P., Hirahara S., Horányi A., Muñoz-Sabater J., Nicolas J., Peubey C., Radu R., Schepers D., Simmons A., Soci C., Abdalla S., Abellan X., Balsamo G., Bechtold P., Biavati G., Bidlot J., Bonavita M., De Chiara G., Dahlgren P., Dee D., Diamantakis M., Dragani R., Flemming J., Forbes R., Fuentes M., Geer A., Haimberger L., Healy S., Hogan R.J., Hólm E., Janisková M., Keeley S., Laloyaux P., Lopez P., Lupu C., Radnoti G., de Rosnay P., Rozum I., Vamborg F., Villaume S., Thépaut J.N. (2020). The ERA5 global reanalysis. Q. J. R. Meteorol. Soc..

[17] A. Jarvis, H.I. Reuter, A. Nelson, E. Guevara, 2008, Hole-fill. SRTM globe version 4 available CGIAR-CSI SRTM 90m database.

[18] Büttner G., Manakos E.I., Braun M. (2014). Land Use and Land Cover Mapping in Europe: Practices & Trends.

[19] Wieder W.R., Boehnert J., Bonan J.B., Langseth M. (2014). Regridded harmonized world soil database v1.2. Oak Ridge Natl. Lab. Distrib. Act. Arch. Cent..

[20] Olivera F., Valenzuela M., Srinivasan R., Choi J., Cho H., Koka S., Agrawal A. (2006). ArcGIS-SWAT: a geodata model and GIS interface for SWAT. J. Am, Water Resour, Assoc..

[21] Gupta H.V., Kling H., Yilmaz K.K., Martinez G.F. (2009). Decomposition of the mean squared error and NSE performance criteria: implications for improving hydrological modelling. J. Hydrol..

[22] Abbaspour K.C., Rouholahnejad E., Vaghefi S., Srinivasan R., Yang H., Kløve B. (2015). A continental-scale hydrology and water quality model for Europe: calibration and uncertainty of a high-resolution large-scale SWAT model. J. Hydrol..

[23] Clavera-Gispert R., Quintana-Seguí P., Palazón L., Zabaleta A., Cenobio O., Barella-Ortiz A., Beguería S. (2023). Streamflow trends of the Pyrenees using observations and multi-model approach (1980–2013). J. Hydrol..

[24] Cenobio-Cruz O., Quintana-Seguí P., Barella-Ortiz A., Zabaleta A., Garrote L., Clavera-Gispert R., Habets F., Beguería S. (2023). Improvement of low flows simulation in the SASER hydrological modeling chain. J. Hydrol. X.

[25] Bonsoms J., López-Moreno J.I., Alonso-González E., Deschamps-Berger C., Oliva M. (2024). Rain-on-snow responses to warmer Pyrenees: a sensitivity analysis using a physically based snow hydrological model. Nat. Hazards Earth Syst. Sci..

