# Supplementary material for: The PIRAGUA_atmos_analysis and PIRAGUA_hydro_analysis: Water balance components data sets for the Pyrenees
Source: Data Brief. 2026 Jul 17;68:113071. doi: 10.1016/j.dib.2026.113071 (PMC13450438; doi:10.1016/j.dib.2026.113071)
Supplement: Supplementary file 1 [file mmc1.docx]

# **Supplementary material**

*Supplementary Table 1. List and characteristics of the selected gauging stations for the hydrological model evaluation process.*

| *Source* | *Official ID* | *Bassin* | *Name* | *UTM X* | *UTM Y* | *Area (km2)* |
| --- | --- | --- | --- | --- | --- | --- |
| *CHC* | *1105* | *Urumea* | *Ereñozu* | *585932* | *4788406* | *215* |
| *CHC* | *1106* | *Bidasoa* | *Endarlaza* | *603040* | *4794358* | *681* |
| *CHC* | *1163* | *Ibaizábal* | *Lemona* | *519908* | *4784351* | *251* |
| *CHE* | *9013* | *Cinca* | *Graus* | *776199* | *4678030* | *893* |
| *CHE* | *9018* | *Aragón* | *Jaca* | *700710* | *4716908* | *238* |
| *CHE* | *9020* | *Segre* | *Puigcerda* | *903720* | *4711072* | *145* |
| *CHE* | *9021* | *Segre* | *Puigcerda* | *905879* | *4708608* | *297* |
| *CHE* | *9022* | *Segre* | *Seo de Urgel* | *866737* | *4699421* | *559* |
| *CHE* | *9023* | *Segre* | *Seo de Urgel* | *867336* | *4698549* | *1233* |
| *CHE* | *9040* | *Cinca* | *Boltaña* | *753206* | *4702793* | *626* |
| *CHE* | *9046* | *Cinca* | *Lecina* | *751209* | *4678709* | *110* |
| *CHE* | *9047* | *Cinca* | *Capella* | *780356* | *4676877* | *426* |
| *CHE* | *9061* | *Aragón* | *Javierregay* | *684303* | *4716256* | *348* |
| *CHE* | *9062* | *Aragón* | *Binies* | *681461* | *4724956* | *161* |
| *CHE* | *9063* | *Aragón* | *Sigües* | *662955* | *4723305* | *506* |
| *CHE* | *9067* | *Arga* | *Olave* | *613772* | *4749604* | *240* |
| *CHE* | *9080* | *Aragón* | *Zuriza* | *678062* | *4747864* | *47* |
| *CHE* | *9085* | *Arga* | *Riezu* | *585541* | *4735265* | *14* |
| *CHE* | *9111* | *Segre* | *Orgaña* | *858440* | *4684390* | *2384* |
| *CHE* | *9135* | *Segre* | *Alins* | *855299* | *4720275* | *60* |
| *CHE* | *9148* | *Segre* | *Coll de nargo* | *855299* | *4677062* | *69* |
| *CHE* | *9198* | *Segre* | *Alins* | *855240* | *4720393* | *83* |
| *CHE* | *9234* | *Aragón* | *Oza* | *687079* | *4743507* | *75* |
| *CHE* | *9256* | *Segre* | *Isobol* | *896406* | *4702383* | *200* |
| *CHE* | *9258* | *Cinca* | *Campo* | *778709* | *4702497* | *540* |
| *CHE* | *9259* | *Aragón* | *Izalzu* | *658739* | *4753066* | *49* |
| *CHE* | *9265* | *Segre* | *Tirvia* | *847746* | *4715949* | *410* |
| *CHE* | *9268* | *Aragón* | *Isaba* | *669316* | *4747262* | *188* |
| *CHE* | *9269* | *Aragón* | *Aragües del Puerto* | *687170* | *4727379* | *75* |
| *CHE* | *9271* | *Aragón* | *Canfranc Antiguo* | *702638* | *4732667* | *101* |
| *GN* | *AN4311* | *Arga* | *Udabe* | *596595* | *4757975* | *78* |
| *GN* | *AN434* | *Arga* | *Iribas* | *589621* | *4760454* | *66* |
| *GN* | *AN439* | *Arga* | *Irurtzun* | *595050* | *4752651* | *253* |
| *GN* | *AN520* | *Aragón* | *Otxagabia* | *655829* | *4754357* | *73* |
| *GN* | *AN532* | *Aragón* | *Sorogain* | *630085* | *4759749* | *24* |
| *GN* | *AN533* | *Aragón* | *Espinal* | *634327* | *4758762* | *47* |
| *GN* | *AN543* | *Aragón* | *Iriberri* | *620020* | *4722118* | *15* |
| *GN* | *AN942* | *Bidasoa* | *Elorriaga* | *606471* | *4776631* | *75* |
| *DFG* | *C7Z1* | *Oria* | *Berastegi* | *577447* | *4776898* | *33* |
| *ACA* | *EA016* | *Fluviá* | *Esponellà* | *978986* | *4685764* | *804* |
| *ACA* | *EA033* | *Ter* | *Ripoll* | *929126* | *4682012* | *737* |
| *ACA* | *EA072* | *Llobregat* | *Sant Joan de les Abadesses* | *932732* | *4687732* | *301* |
| *ACA* | *EA078* | *Ter* | *Guardiola de Berguedà* | *901988* | *4685072* | *354* |
| *DFB* | *NB01* | *Ibaizábal* | *Saratxo* | *499783* | *4764591* | *88* |
| *EAUFR.* | *O0010040* | *Garonne* | *La Garonne à Saint-Béat* | *801330* | *4758035* | *647* |
| *EAUFR.* | *O0015310* | *Garonne* | *Le Maudan à Fos* | *806170* | *4752845* | *36* |
| *EAUFR.* | *O0105110* | *Garonne* | *La Neste de Cap de Long à Aragnouet* | *754923* | *4745508* | *5* |
| *EAUFR.* | *O0126210* | *Garonne* | *La Neste de Rioumajou à Tramezaïgues* | *769892* | *4742983* | *67* |
| *EAUFR.* | *O0362510* | *Garonne* | *Le Salat à Soueix-Rogalle* | *843985* | *4759294* | *385* |
| *EAUFR.* | *O0384010* | *Garonne* | *L'Arac à Soulan* | *845551* | *4758294* | *170* |
| *EAUFR.* | *O0485110* | *Garonne* | *Le Lachein à Balaguères* | *828784* | *4763717* | *9* |
| *EAUFR.* | *O0554010* | *Garonne* | *L'Arbas à Castelbiague* | *819946* | *4773455* | *92* |
| *EAUFR.* | *O0584310* | *Garonne* | *Le Lens à Betchat* | *829047* | *4779751* | *61* |
| *EAUFR.* | *O0624010* | *Garonne* | *Le Volp à Montberaud* | *836806* | *4785160* | *90* |
| *EAUFR.* | *O0744030* | *Garonne* | *L'Arize au Mas-d'Azil* | *855006* | *4779328* | *220* |
| *EAUFR.* | *O1076010* | *Garonne* | *Le Quioulès à Château-Verdun* | *882050* | *4738615* | *54* |
| *EAUFR.* | *O1115010* | *Garonne* | *L'Artigue à Auzat* | *861669* | *4738010* | *24* |
| *EAUFR.* | *O1432930* | *Garonne* | *L'Hers à Bélesta* | *902264* | *4760733* | *134* |
| *EAUFR.* | *O1442910* | *Garonne* | *L'Hers Vif au Peyrat* | *902628* | *4767567* | *191* |
| *EAUFR.* | *O1464010* | *Garonne* | *Le Blau à Chalabre* | *908946* | *4770575* | *77* |
| *EAUFR.* | *O1484310* | *Garonne* | *La Touyre à Lavelanet* | *895842* | *4765630* | *68* |
| *EAUFR.* | *O1584610* | *Garonne* | *Le Douctouyre à Dun* | *888823* | *4776698* | *136* |
| *EAUFR.* | *Q0115710* | *Adour* | *L'Oussouet à Trébons* | *752562* | *4775467* | *32* |
| *EAUFR.* | *Q0214010* | *Adour* | *L'Échez à Louey* | *745651* | *4784719* | *78* |
| *EAUFR.* | *Q0522520* | *Adour* | *L'Arros à Gourgue* | *765135* | *4780593* | *183* |
| *EAUFR.* | *Q0554010* | *Adour* | *L'Arret-Darre à Vielle-Adour* | *756388* | *4781221* | *12* |
| *EAUFR.* | *Q4801010* | *Adour* | *Le Gave de Pau à Saint-Pé-de-Bigorre* | *732506* | *4776151* | *1123* |
| *EAUFR.* | *Q5501010* | *Adour* | *Le Gave de Pau à Bérenx* | *673508* | *4819478* | *2592* |
| *EAUFR.* | *Q6142910* | *Adour* | *Le Gave d'Ossau à Oloron-Sainte-Marie* | *695338* | *4784506* | *493* |
| *EAUFR.* | *Q6332510* | *Adour* | *Le Gave d'Aspe à Bedous* | *694893* | *4771572* | *433* |
| *EAUFR.* | *Q7002910* | *Adour* | *Le Gave d'Oloron à Oloron-Sainte-Marie* | *694363* | *4785641* | *1101* |
| *EAUFR.* | *Q7322510* | *Adour* | *Le Saison à Mauléon-Licharre* | *672539* | *4790398* | *498* |
| *EAUFR.* | *Q7412910* | *Adour* | *Le Gave d'Oloron à Escos* | *662330* | *4811530* | *2484* |
| *EAUFR.* | *Q8032510* | *Adour* | *La Bidouze à Aïcirits-Camou-Suhast* | *659811* | *4799756* | *246* |
| *EAUFR.* | *Q8345910* | *Adour* | *Le Mendialçu à Hasparren* | *636863* | *4807831* | *17* |
| *EAUFR.* | *Q9164610* | *Adour* | *La Nive des Aldudes à Saint-Étienne-de-Baïgorry* | *635145* | *4782608* | *157* |
| *EAUFR.* | *Y0245210* | *Tech* | *Le Mondony à Amélie-les-Bains-Palalda* | *966109* | *4717306* | *32* |
| *EAUFR.* | *Y0254050* | *Tech* | *Le Tech à Reynès* | *970996* | *4720818* | *477* |
| *EAUFR.* | *Y0255020* | *Tech* | *L'Ample à Reynès* | *968315* | *4720450* | *49* |
| *EAUFR.* | *Y0436420* | *Agly* | *Le Cady à Villefranche-de-Conflent* | *940639* | *4728922* | *60* |
| *EAUFR.* | *Y0624020* | *Agly* | *L'Agly à Saint-Paul-de-Fenouillet* | *949600* | *4753529* | *218* |
| *EAUFR.* | *Y0655010* | *Agly* | *Le Verdouble à Tautavel* | *969695* | *4755802* | *309* |
| *EAUFR.* | *Y1012010* | *Aude* | *L'Aude à Puyvalador* | *920162* | *4734435* | *139* |
| *EAUFR.* | *Y1105010* | *Aude* | *Le Rebenty à Saint-Martin-Lys* | *927356* | *4753620* | *227* |
| *EAUFR.* | *Y1225010* | *Aude* | *Le Lauquet à Greffeil* | *939697* | *4781655* | *66* |
| *EAUFR.* | *Y1232010* | *Aude* | *L'Aude à Carcassonne* | *935420* | *4798418* | *1829* |
| *EAUFR.* | *Y1564010* | *Aude* | *L'Orbieu à Luc-sur-Orbieu* | *969777* | *4797202* | *590* |

*Supplementary Figure S1. Location of gauging stations with natural or quasi-natural flow regimes selected for model validation, and the corresponding upstream catchments. These sites were chosen to minimize the influence of reservoirs, abstractions, or other human alterations, thereby providing the best available approximation of baseline hydrological conditions against which model simulations could be evaluated.*
